# Maternally Inherited Diabetes and Deafness (MIDD) – Atypical Clinical Diabetes Features Leading to the Diagnosis

**DOI:** 10.1210/jcemcr/luad047

**Published:** 2023-05-23

**Authors:** Anna Kyriakidou, Marilena Hadjivassiliou, Anastasia Papapostolou, Michalis K Picolos

**Affiliations:** School of Clinical Medicine, University of Cambridge, Cambridge CB2 0SP, UK; Department of Cardiovascular Genetics and the Laboratory of Forensic Genetics, The Cyprus Institute of Neurology and Genetics, Nicosia 2371, Cyprus; Alithias Endocrinology Center, Nicosia 2324, Cyprus; Alithias Endocrinology Center, Nicosia 2324, Cyprus

**Keywords:** MIDD, mitochondrial diabetes, mitochondrial DNA, insulin-requiring diabetes, heteroplasmy, Cyprus

## Abstract

Maternally inherited diabetes and deafness (MIDD) syndrome refers to a rarely diagnosed disorder caused by pathogenic variants in mtDNA. It was first identified in 1992 and, to date, is considered underdiagnosed because of misclassification to type 1 or type 2 diabetes mellitus. MIDD reflects a multisystem metabolic syndrome commonly resulting in insulin-requiring diabetes and sensorineural deafness but can also lead to a broad range of other manifestations. The spectrum of pathology differs among individuals, likely because of varied degrees of heteroplasmy associated with mtDNA. Heteroplasmy also creates diagnostic difficulties, with a high index of suspicion required to diagnose MIDD in some cases. Here, we review a patient with MIDD who presented with an atypical clinical diabetes picture, additionally documenting his pedigree. To our knowledge, this is the first Cypriot reported with MIDD.

## Introduction

Maternally inherited diabetes and deafness (MIDD) is a syndromic disorder of mitochondria caused by mutations in mtDNA. There is a broad phenotypic expression of the syndrome, even between individuals of the same family, depending on the degree of mutant mtDNA in cells. It is postulated that some people may carry a degree of mutated mtDNA without experiencing any symptomatology if they do not meet the heteroplasmic threshold. However, common presentations include atypical diabetes mellitus (DM) and progressive symmetrical sensorineural hearing impairment. The estimated mean age of onset of diabetes is 37.9 years but can present at any age [1]. Initial management with oral hypoglycemics is usually sufficient in clinical practice, although most patients progress to insulin therapy within 10 years of diagnosis because of the decline in pancreatic β-cell mass.

The most common mutation identified in MIDD is A > G at nucleotide 3243 of mtDNA (m.3243 A > G), located within the tRNA^Leu(UUR)^ gene. More than 85% of people with this mutation develop diabetes associated with MIDD [2]. Other, rarer mutations have also been identified.

MIDD is characterized by uniparental maternal inheritance in line with mtDNA inheritance. Maternal mtDNA inheritance results in the observed heteroplasmic genotype that poses potential diagnostic obstacles in addition to contributing to a diverse phenotypic expression.

## Case Presentation

Our patient is a Caucasian Greek-Cypriot male initially diagnosed with DM at the age 29 years and presenting to our outpatient clinic at aged 30 years. His body mass index at the time of presentation was 16.5 kg/m^2^, with a weight of 47 kg. His diabetes regimen consisted of metformin and repaglinide. Despite normal fasting blood sugars (80-100 mg/dL or 4.4-5.5 mmol/L), his postprandial readings averaged 200 to 260 mg/dL (11.1-14.4 mmol/L). This had led to avoidance of carbohydrate consumption with subsequent weight loss. His medical history included right brachial plexus palsy since birth, hyperlipidemia, and long-standing trigeminal neuralgia. He was a nonsmoker.

**Figure 1.**
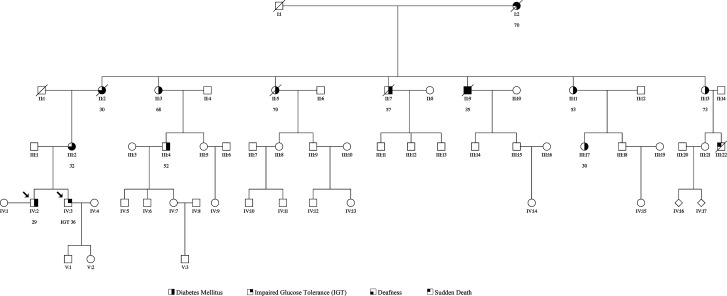
Family pedigree of index patient.

Family history revealed diabetes in multiple family members, hearing impairment in the mother and maternal grandmother, and maternal cardiomyopathy. Both the mother and maternal grandmother had died in their 60s with “multiorgan failure.” Figure 1 illustrates the family pedigree diagram of the patient.

## Diagnostic Assessment

Investigations performed at presentation included antibody testing for glutamic acid decarboxylase-65 and islet cell antibodies), which were negative. His C-peptide levels were 3.52 ng/mL (1.17 nmol/L) with a glucose value of 314 mg/dL (17.4 mmol/L). His hemoglobin A1c was 6.3%. The rest of the laboratory workup revealed microalbuminuria with a random urine microalbumin/creatine ratio of 197.4 mg/g (22.3 mg/mmol) (normal = <30 mg/g or <3.4 mg/mmol). Additional investigations included molecular genetic testing. This was performed by polymerase chain reaction and direct sequencing for confirmation, looking for the m.3243 A > G mutation. The test was positive, with an intermediate level of mutation identified (ie, 30%-70% heteroplasmy in peripheral lymphocyte DNA extracted from the blood sample used for analysis). This confirmed the diagnosis of MIDD.

Audiometry assessment revealed bilateral mild hearing impairment at high-frequency sounds. Fundoscopy and cardiology evaluation including 2-dimensional echocardiography did not reveal abnormalities.

After genetic counselling and family testing, his brother was found to have impaired glucose tolerance at aged 36 years.

## Treatment

Management implemented included a basal-bolus insulin regimen with discontinuation of oral hypoglycemics. The patient’s statin medication was continued, and he was also prescribed daily telmisartan 40 mg for renoprotection and coenzyme Q10. His new insulin regimen led to more liberal food intake and improvement of his glucose profile (fasting blood glucose of approximately 80**-**99 mg/dL [4.4-5.5 mmol/L]; 2-hour postprandial readings in the range of 100-160 mg/dL [5.5-8.9 mmol/L]). His hemoglobin A1c dropped to 5.9% 4 months later, and his weight increased by about 5 kg within a few months. His urine microalbumin/creatinine ratio improved but remained elevated at 98 mg/g.

## Outcome and Follow-up

Monitoring involved regular cardiology, ear, nose, and throat, and ophthalmology reviews. At the age of 34 years, following neurological evaluation for headaches, magnetic resonance imaging T2/fluid-attenuated inversion recovery of the brain was performed. This identified widespread periventricular and subcortical nonspecific white matter changes. Magnetic resonance angiography showed a large venous angioma in the cerebellum and brainstem and a suggestion of cavernoma in the pons area.

## Discussion

This was the first kindred with MIDD identified in Cyprus. The prevalence of MIDD is estimated approximately 0.5% to 2.8% of patients with type 2 DM [3]. However, epidemiological studies for mtDNA diseases are greatly complicated by the heteroplasmic phenomenon. Cells not satisfying the heteroplasmic threshold of pathology result in a subclinical presentation despite possessing the pathogenic variant. Such cases might thus not be identified on a population level, meaning that disease prevalence becomes underestimated. Reported prevalence of MIDD in the literature also varies depending on the sensitivity of the testing method used, the sample of patients, and patient ethnic groups. Nonetheless, to our knowledge, the highest number of MIDD patients have been reported in Asia with 108 cases, followed by Europe with 43 cases, and the United States with 8 cases [4].

The proband's diabetes presentation was atypical because his C-peptide levels were within normal range while hyperglycemic, glutamic acid decarboxylase and islet cell antibodies were negative, which is inconsistent with a diagnosis of type 1 DM or latent autoimmune diabetes of adults. His low body mass index and relatively young age were also inconsistent with type 2 DM; however, he seemed to be responding partially to oral hypoglycemics given his normal fasting sugars. This atypical presentation, in combination with the strong family history of diabetes, prompted investigation for rarer monogenic causes.

Family history identified 24 relatives of maternal descendance across 5 generations. Twelve of 24 relatives have been diagnosed with DM (50%) and 1/24 with impaired glucose tolerance (4.17%). Of note, 3/24 (12.5%) sudden deaths have been identified in 3 consecutive generations (individuals I2, II9, and III22; Fig. 1), 1 aged 70 years, 1 aged 35 years, and another at an unknown age. Of these, 2 were known to have diabetes.

Prevalence of hearing impairment was less than that of DM, with 3/24 individuals being diagnosed (12.5%). There is limited literature available on the incidence rates of diabetes and sensorineural deafness within affected families, and those reported exhibit great discrepancy. This may be largely influenced by the limited family tracing possible with very few relatives of known medical history being described.

Following diagnosis, initiation of insulin normalized the patient’s glucose profile, in line with the evidence for progressive insulin deficiency characteristic of MIDD. As such, all oral hypoglycemics were withheld, including metformin because of the potential risk of lactic acidosis. Coenzyme Q10 was prescribed because of its antioxidant nature and its effects in the mitochondria as a respiratory chain cofactor, despite the limited, high-quality evidence supporting its use [5].

The patient developed manifestations of proteinuria early on during disease presentation. This probably suggests that renal pathology preceded diabetic complications and was not directly related to diabetic nephropathy. In support of this, the most common renal pathology identified in MIDD cases is focal segmental glomerulosclerosis, which very commonly manifests as proteinuria in early adulthood [6].

MIDD has been associated with brain abnormalities, including a decline in brain function and cognitive impairment compared with controls. Both structural magnetic resonance imaging studies and cognitive assessments have been used to provide evidence of cerebellar atrophy and basal ganglia calcification. Cognitively, patients seem to perform more poorly in sustained attention, verbal working memory, and abstract reasoning compared with controls. Some patients also display functional defects and/or neuronal loss in the vermis on hydrogen magnetic resonance spectrometry [7]. However, studies on the impact of MIDD on the nervous system are sparse. To date, our patient has not yet developed any cognitive impairments or clinical features of neural pathology, despite the structural brain abnormalities discussed. To our knowledge, the white matter changes seen have not been described in other patients with MIDD in the literature, possibly reflecting the diverse phenotypic expression of the syndrome. Hence, their clinical significance remains to be determined by close long-term monitoring.

mtDNA adopts uniparental maternal inheritance patterns in multiple eukaryotic species, including humans. This likely reflects an evolutionary attempt to avoid heteroplasmy between potentially distant mtDNA haplotypes, with maternal inheritance being the safest option because of reduced exposure of oocytes to reactive oxygen species compared with sperm [8].

It thus follows that any mutant maternal mtDNA is transmitted to the embryos with no potential salvage by paternal inheritance. In cases of pathogenic variants, the extent of pathology vertically transmitted to the embryos depends on the percentage of mutated circular mtDNA molecules in the mother and the bottleneck effect whereby only a small proportion of maternal mitochondrial genomes are passed on to the offspring by random selection. This gives rise to the heteroplasmy phenomenon; a mosaicism of both mutant and wild-type mtDNA molecules is generated with variable expressivity across cells of the same individual but also between siblings. A threshold is hypothesized to exist, referring to a level up to which cells can tolerate defective mtDNA molecules, with different cells having different thresholds according to their energy demands [9]. Exceeding this threshold can result in metabolic dysfunction and associated clinical syndromes.

In the case of MIDD, the maternal inheritance phenotype is associated with genotypic point mutations in mtDNA with the degree of heteroplasmy influencing the extent of pathology. This has implications for genetic counselling in siblings of affected individuals because unless genetic testing is performed and the level of heteroplasmy determined, pathology in siblings cannot be predicted merely based on the phenotype of the affected individual. It also poses diagnostic difficulties because some tissues may not express pathogenic variants in heteroplasmic levels that satisfy the threshold for diagnosis, giving false-negative outcomes. As such, careful selection of samples for genetic diagnosis is essential. Muscle tissues seem to express high levels of mutant mtDNA and could be a safe option for diagnosis if blood lymphocyte DNA samples are not fertile while there is still diagnostic doubt.

Early diagnosis is important for optimal early management and appropriate follow-up because of the broad spectrum of possible manifestations.

## Learning Points

- Atypical diabetes features, strong family history, and negative antibodies should prompt evaluation for monogenic causes of diabetes.
- In maternally inherited diabetes and deafness (MIDD), diabetes is the most prevalent pathology, followed by hearing impairment.
- Basal-bolus insulin was able to adequately control our patient's diabetes.
- Microalbuminuria, if present early, is most likely a feature of MIDD and does not usually represent a diabetic complication.
- Heteroplasmy results in a diverse phenotype; hence, diagnosis relies heavily on a high index of suspicion.
- The most important diagnostic investigation is genetic testing of appropriate tissue samples.

## Data Availability

Original data generated and analyzed during this study are included in this published article.

## Abbreviations

DM: diabetes mellitus
MIDD: maternally inherited diabetes and deafness
